# Cuproptosis meets sonodynamics: a nanomedicine platform for multimodal cancer therapy

**DOI:** 10.1039/d6ra01867j

**Published:** 2026-04-16

**Authors:** Jun-Jie Zhou, Yan-Chuan Feng, Chun-Yan Song, Yu-Ting Fan, Guo-Qing Wang, Xi-Bo Zhao

**Affiliations:** a The Stomatological Hospital, Anyang Sixth People's Hospital Anyang 45500 China zhoujunjie@whu.edu.cn zhaoxibo1220@163.com; b Anyang Key Laboratory of Digital Stomatology China

## Abstract

Cancer treatment is shifting beyond the traditional single-agent therapy. The focus of research has shifted to combination strategies that target specific metabolic dependencies in the tumor microenvironment (TME). Recently, cuproptosis has been characterized as a type of regulated cell death triggered by copper overload and the resulting mitochondrial stress. This novel mechanism presents a promising strategy for treating cancer by focusing on the cells' dependence on enhanced metabolic processes. This approach has faced two significant challenges in its clinical translation. The delivery of copper ions to tumor sites is insufficient, and tumors have patient-specific sensitivity to cell death triggered by copper. Sonodynamic therapy (SDT) provides unique benefits, including the ability to penetrate deep tissues and control treatment in both space and time. Nonetheless, its treatment efficacy is frequently reduced by the low-oxygen tumor environment and strong intracellular antioxidant defenses. In this article, we systematically explore the synergistic mechanisms between SDT and cuproptosis. Significantly, we point out that thoughtfully designed nanomedicines function as the key connection to promote this strategic combination. Specifically, ultrasound (US) prompts the controlled release of copper from nanomedicines and simultaneously creates reactive oxygen species (ROS). The ROS reduce intracellular glutathione (GSH) levels, which decreases the cell's defense threshold and makes cancer cells more susceptible to copper-induced cell death. Meanwhile, the copper ions that are released participate in Fenton-like reactions, the core mechanism of chemodynamic therapy (CDT). This leads to the production of more ROS, which in turn increases the oxidative stress caused by SDT. This review offers a critical examination of the therapeutic potential linked to the synergy of SDT-induced cuproptosis. Our focus is on how this synergistic approach not only overcomes multidrug resistance but also triggers strong immunogenic cell death (ICD) when combined with chemotherapy, immunotherapy, and metabolic strategies. In conclusion, we summarize the major challenges in translating to clinical practice, especially regarding large-scale production and biosafety. Looking forward, we suggest developing intelligent nanomedicines that are responsive to the tumor microenvironment, aiming for precise and highly effective cancer therapy.

## Abstract 2

Application of SDT-induced cuproptosis nanomedicines combined with chemotherapy, photothermal therapy, immunotherapy, ferroptosis, gas therapy, and metabolic therapy.

## Graphical abstract

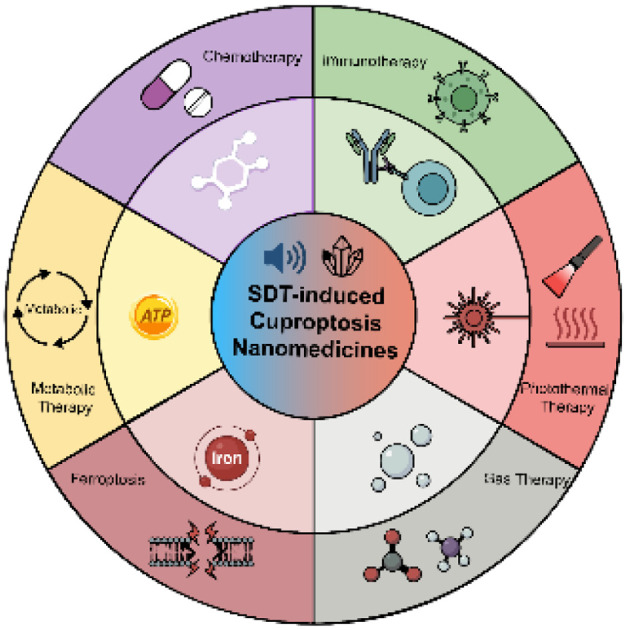

## 1. Introduction

The treatment landscape in oncology is experiencing a major transformation.^1–4^ There has been significant progress in the development of cancer treatment methods. Due to a deeper understanding of the tumor microenvironment (TME), oncology treatment strategies have moved from single methods to integrated, multimodal approaches.^5,6^ While chemotherapy, radiotherapy, and surgical removal are key to cancer treatment, their clinical success is often diminished by several difficulties.^7,8^ Current cancer therapies are limited by multidrug resistance, significant systemic toxicity, and the ongoing challenge of targeting dormant cancer stem cells.^9–11^ Due to these limitations, novel therapeutic approaches are being developed continuously.^12–14^ Among the innovative techniques, cuproptosis and sonodynamic therapy (SDT) have attracted notable interest.^15–17^ Their unique therapeutic advantages and potential for clinical application are the core reasons for this research focus.^18,19^

SDT is recognized as a promising non-invasive therapy that uses ultrasound to stimulate sonosensitizers, which in turn generate reactive oxygen species (ROS) to kill cancer cells.^19–21^ Because of its ability to deeply penetrate tissues, accurately target specific areas, and exhibit a more favorable side-effect profile than traditional treatments such as photodynamic therapy (PDT), this technique has drawn considerable interest.^22–24^ The development of nanotechnology has advanced SDT, improving both its therapeutic efficacy and targeting precision.^25–27^

Cuproptosis is a newly discovered form of regulated cell death. It is entirely dependent on the intracellular buildup of copper and the functionality of mitochondrial respiration.^28–31^ The process of cuproptosis is triggered by the direct attachment of copper ions, especially the toxic cuprous ion (Cu^+^), to the lipoylated elements of the mitochondrial tricarboxylic acid (TCA) cycle.^31–33^ Ferredoxin 1 (FDX1) plays a crucial role in this process by converting Cu^2+^ to Cu^+^ and facilitating the lipoylation of important enzymes such as dihydrolipoamide *S*-acetyltransferase (DLAT).^34–36^ The binding of Cu^+^ to lipoylated DLAT triggers its abnormal oligomerization, leading to proteotoxic stress. At the same time, this process leads to a reduction in iron–sulfur cluster proteins like lipoyl synthase (LIAS).^34–36^ Together, these events result in the loss of Fe–S cluster proteins, disruption of mitochondrial metabolism, and ultimately, cell death.^32,33,37^ Notably, cancer cells exhibit an increased demand for copper and copper dysregulation compared to normal cells. This specific metabolic property may increase their responsiveness to therapies that induce cuproptosis.^28,38^ The targeted susceptibility presents a promising chance for therapy. In 2022, Tsvetkov's team confirmed that the mitochondrial TCA cycle is the main center of cuproptosis.^29^ Clinically, this finding supports the advancement of predictive biomarkers for selecting patients for cuproptosis-related therapies.^39,40^ From a therapeutic design perspective, it provides a framework for developing nanomedicines that effectively overcome these shortcomings.

Cuproptosis and SDT each exhibit potential in tumor treatment, yet they face different limitations.^41^ For example, the effectiveness of SDT is frequently limited by the low oxygen levels in the TME and elevated glutathione (GSH) concentrations.^42,43^ In contrast, cuproptosis-based treatment alone may face challenges such as inefficient copper ion delivery and heterogeneous sensitivity among tumor cells.^39,44^ Consequently, synergistic therapeutic strategies have emerged, aiming to overcome the shortcomings of individual modalities and achieve an enhanced therapeutic outcome. Such synergy not only enhances direct tumor cell killing but also modulates the TME and induces immunogenic cell death (ICD).^45^ This, in turn, activates the host's antitumor immune response for more effective control of tumor growth and metastasis.

The combination of SDT and cuproptosis provides a promising multi-modal strategy for cancer therapy.^46,47^ Based on a nanomedicine platform, this approach presents a synergistic strategy with notable potential to overcome resistance mechanisms.^48,49^ Nanomedicines act as ideal carriers for the simultaneous delivery of sonosensitizers and copper sources, providing controlled release, increased tumor accumulation through the enhanced permeability and retention (EPR) effect, and activation that is responsive to the TME.^50,51^ ROS generated during SDT can enhance the oxidative environment, potentially promoting the release of copper ions from nanocarriers and increasing cellular sensitivity to copper toxicity.^52,53^ On the other hand, copper ions can produce more ROS through Fenton-like reactions, which regulates the cellular redox balance and makes cells more sensitive to SDT.^54,55^ Furthermore, typical characteristics of the TME, such as low oxygen, acidic environments, and high GSH levels, often weaken the effectiveness of conventional treatments like SDT.^43,56^ Therefore, copper-based nanomedicines can use these characteristics to transform them into therapeutic benefits.^57,58^ These TME conditions, for instance, can lead to copper release, trigger CDT, and affect the induction of cuproptosis, thereby converting therapeutic barriers into opportunities and forming a self-amplifying cytotoxic cycle.^41,59,60^ As a result, this intricate interaction transforms the limitations of the TME into favorable conditions for therapy.

**Fig. 1:**
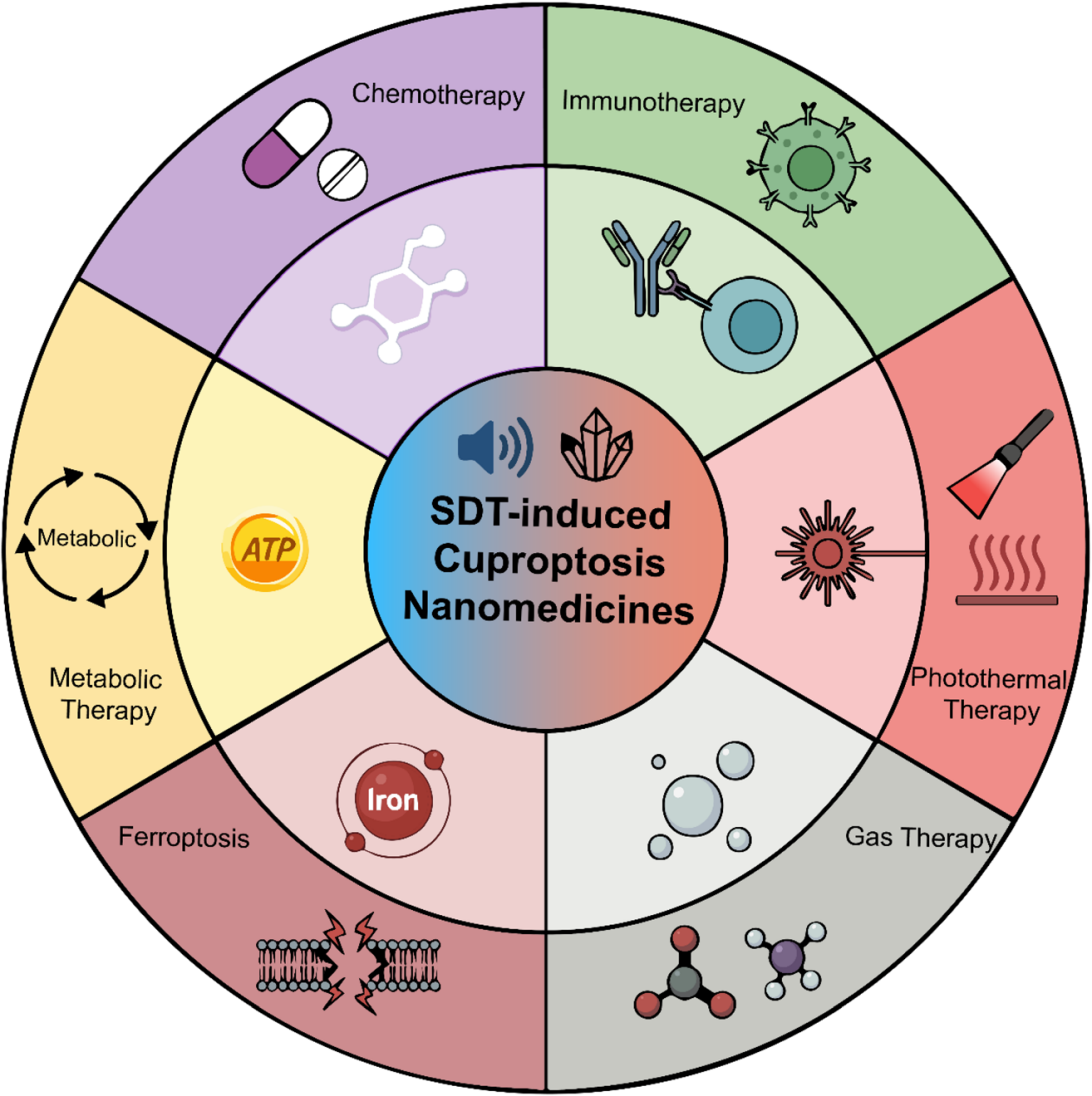
The illustration shows the application of SDT-induced cuproptosis nanomedicines combined with chemotherapy, photothermal therapy, immunotherapy, ferroptosis, gas therapy, and metabolic therapy.

**Fig. 2:**
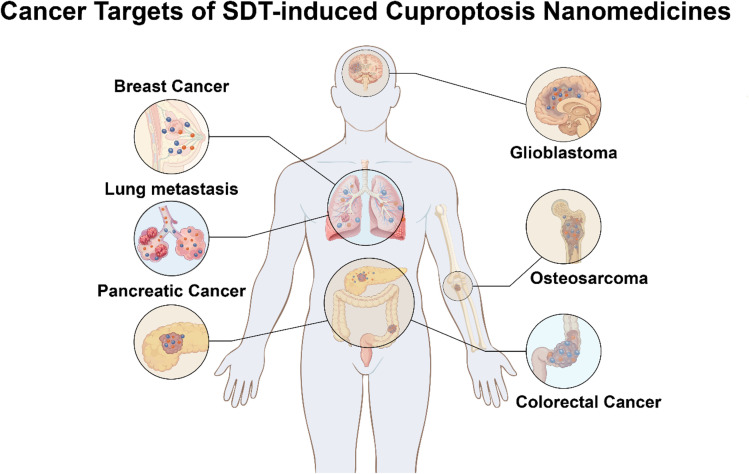
The scheme demonstrates the application of SDT-induced cuproptosis nanomedicines in the various tumors.

This review provides a mechanistic-centric analysis of the SDT–cuproptosis synergy. We systematically deconstruct how nanomedicines function as the operational bridge: (1) US acts as a spatiotemporal trigger for controlled copper ion release. (2) US-generated ROS deplete GSH, lowering the cellular defense threshold and sensitizing cancer cells to copper-induced death. (3) The released copper ions, particularly Cu^+^, engage in Fenton-like reactions that further amplify ROS production, creating a self-reinforcing cytotoxic loop. Building on this mechanistic foundation, we critically evaluate how this synergy is leveraged in combination with chemotherapy, immunotherapy, photothermal therapy (PTT), gas therapy, ferroptosis, and metabolic interventions (Fig. 1). The discussion is divided by tumor types, such as breast cancer, glioblastoma, colorectal cancer, pancreatic cancer, lymphoma, osteosarcoma, and others. This provides an organized reference for clinical translation in this field (Fig. 2).

## 2. Core mechanisms of US-triggered cuproptosis: from ion release to redox sensitization

**Fig. 3:**
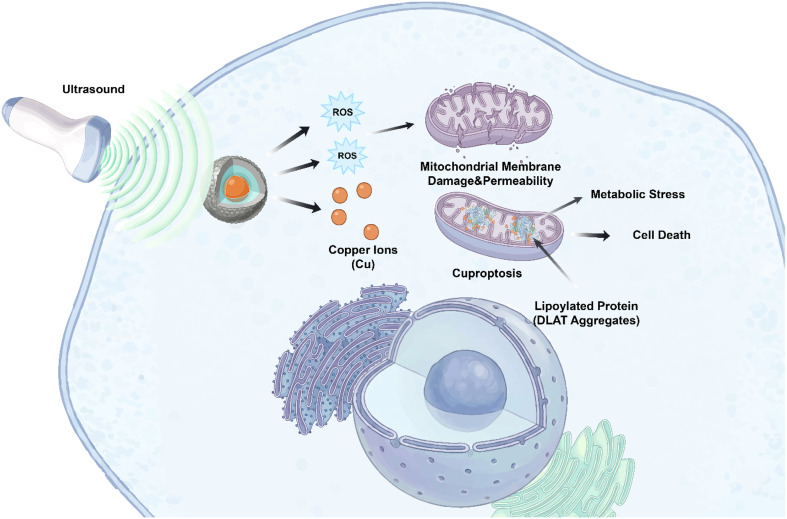
The scheme illustrates the mechanism of sonodynamic-cuproptosis nanomedicines.

US can trigger cuproptosis through two mechanisms: the direct physical release of copper ions from nanocarriers, and the indirect modulation of the intracellular redox environment *via* sonosensitizer-generated ROS.^52^ Using US to manage copper-dependent cell death with spatial and temporal precision is a major benefit.^61–63^ It can target activation in the TME and lowering systemic copper toxicity. This section dissects these core mechanisms, emphasizing how nanomedicine design dictates the efficiency and selectivity of the SDT–cuproptosis synergy (Fig. 3).

### 2.1 US-triggered copper ion release and cuproptosis

**Fig. 4:**
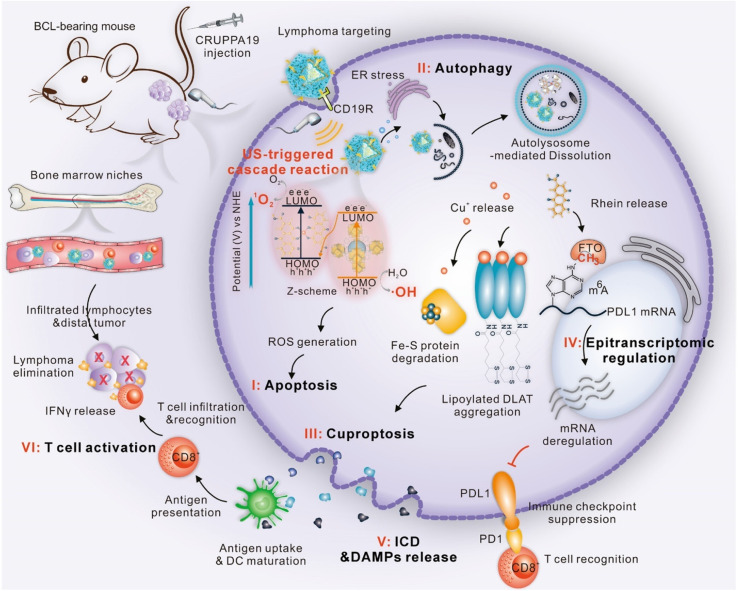
Schematic illustration of the synthesis of CRUPPA19. Reprinted with permission from ref. 66. Copyright 2025 American Chemical Society.

US generates physical effects such as mechanical cavitation, localized heating, and the activation of sonosensitizers bound to surfaces. These effects can help in achieving a controlled manner of release. In particular, these effects allow for the controlled release of copper ions from nanocarriers.^47,64,65^ After being released, the copper accumulates intracellularly, reaching a high level that triggers cuproptosis. Fei Yan and his team designed an epigenetic nanosensitizer (CRUPPA19) based on a UiO-66 metal–organic framework (Fig. 4).^66^ The system is composed of UiO-66–NH_2_, which contains a copper complex of an m6A-mRNA demethylase inhibitor, and is modified with an anti-CD19 antibody. This nanoplatform showed outstanding capability for the precise, ultrasound-regulated release of various therapeutic agents. It allowed targeted treatment of B-cell lymphoma tissues and infiltrating lymphoma cells in the bone marrow. The release of Cu and Rhein from CRUPPA19 was triggered by US stimulation through autophagy. The Cu^2+^ ions that were released disrupted the mitochondrial TCA cycle, causing abnormal oligomerization of lipoylated proteins and a loss of Fe–S cluster proteins, which in turn triggered cuproptosis. This work demonstrated that US not only triggered release but also initiated a cascade involving apoptosis, autophagy, and ultimately, cuproptosis, highlighting the multifactorial impact of US. *In vitro* studies revealed that the CRUPPA19+US combination significantly killed B-cell lymphoma cells. The treatment triggered high levels of intracellular ROS and an apoptosis rate of about 70%, while having minimal adverse effects on normal cells. *In vivo*, the CRUPPA19+US treatment effectively reduced tumors in mice bearing B-cell lymphoma. The treatment not only removed primary and metastatic lymphoma but also cleared lymphoma cells from the bone marrow, demonstrating significantly greater efficacy than either CRUPPA19 or US alone. The CRUPPA19+US treatment group showed downregulation of anti-apoptotic genes and upregulation of pro-apoptotic genes. At the same time, there were significant changes in the expression of genes associated with copper (like FDX1 and DLAT) and immune checkpoint-related genes (such as PD-L1). The findings suggest that CRUPPA19+US works through an ultrasound-triggered cascade that involves: (I) the induction of apoptosis; (II) the initiation of autophagy; (III) the enhancement of cuproptosis; (IV) the induction of immunogenic cell death; and (V) the activation of T-cells. This approach, characterized by the convergence of apoptosis, cuproptosis, and immune activation, successfully kills B-cell lymphoma cells and offers a novel treatment strategy for hematological malignancies.

### 2.2 Sonodynamic generation of ROS influences the regulation of cuproptosis

**Fig. 5:**
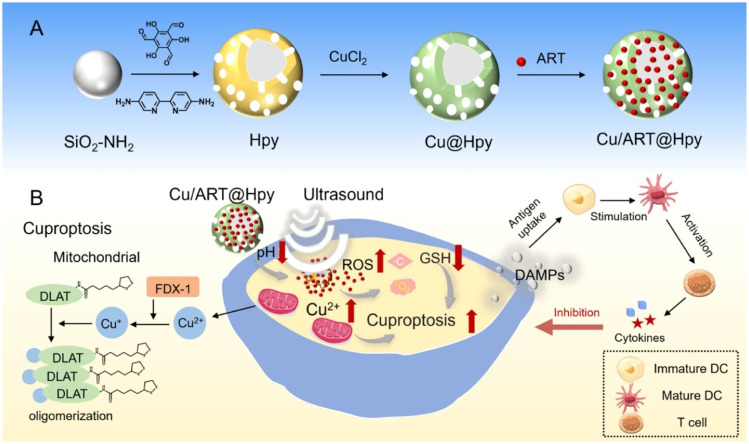
Schematic illustration of the Cu/ART@Hpy. (A) The preparation of Cu/ART@Hpy. (B) The representation of Cu/ART@Hpy for enhancing cuproptosis through sonodynamics and cooperative cancer therapy. Reprinted with permission from ref. 68. Copyright 2025 Elsevier.

ROS, the primary chemical product of SDT, can indirectly potentiates cuproptosis by depleting intracellular GSH and disrupting copper homeostasis.^67^ Li *et al.* developed a multifunctional hybrid nanoparticle based on a porous organic polymer (Cu/ART@Hpy), which realized a pH-responsive release of Cu^2+^ in the acidic tumor microenvironment (Fig. 5).^68^ This system was constructed from a bipyridine-based porous organic polymer (POP) synthesized using aminated silica as a template, subsequently coordinated with copper ions and loaded with the sonosensitizer artesunate (ART). This nanoplatform exhibits excellent performance for the precise, TME-responsive and ultrasound-controlled release of multiple therapeutics. As a significant intracellular antioxidant, GSH also acts to chelate copper. Cu^2+^ ions from Cu/ART@Hpy are converted to Cu^+^ by GSH within cells. Through a Fenton-like process, Cu^+^ ions convert internal H_2_O_2_ into the cytotoxic ˙OH. This process reduces GSH levels at the same time, which increases oxidative stress. The findings indicated that ˙OH generation is greatly enhanced in the tumor microenvironment and depends on GSH. The release of Cu^2+^ ions and the subsequent generation of Cu^+^ ions disrupt the mitochondrial TCA cycle, causing abnormal oligomerization of lipoylated proteins and a decrease in Fe–S cluster proteins, which induces cuproptosis. The design demonstrates how SDT-generated ROS function as a metabolic sensitizer, transforming the tumor's antioxidant defenses into a weakness that amplifies copper toxicity. *In vitro* studies revealed that the Cu/ART@Hpy and ultrasound combination (Cu/ART@Hpy+US) significantly killed 4T1 breast cancer cells. The treatment resulted in high intracellular ROS levels and a cell death rate of about 70%, while being minimally toxic to normal cells. In 4T1 tumor-bearing mice, the Cu/ART@Hpy+US treatment significantly suppressed tumors, showing much better results than either Cu/ART@Hpy or US alone. The Cu/ART@Hpy+US treatment group showed a decrease in anti-apoptotic gene expression and an increase in pro-apoptotic gene expression, along with notable changes in copper-related gene expression within the tumor tissues. These results indicate that Cu/ART@Hpy+US, through the combined effect of SDT-enhanced cuproptosis, not only directly kills tumor cells but also induces cell death *via* multiple pathways including apoptosis and cuproptosis, providing an innovative strategy for cancer therapy.

## 3. Sonodynamic nanomedicines for the combined cuproptosis induction with chemotherapy and CDT

### 3.1 Principles of CDT and its synergy with SDT-induced cuproptosis

CDT is an emerging cancer treatment modality that employs chemical reactions—primarily Fenton or Fenton-like reactions—occurring *in situ* within the TME to generate highly cytotoxic ROS, such as hydroxyl radicals (˙OH).^69–72^ This process typically involves transition-metal ions catalyzing the decomposition of endogenous or exogenous hydrogen peroxide (H_2_O_2_), which is often increased in tumor cells.^73–75^ Copper ions released from SDT–cuproptosis nanomedicines serve a dual function: they are both the direct inducers of cuproptosis and highly efficient catalysts for Fenton-like reactions.^76^

The integration of SDT, cuproptosis, and CDT operates through several interconnected pathways: firstly, SDT generates primary ROS and may enhance the release or activation of copper ions from the nanomedicine.^47,63,77,78^ Then the released copper ions induce cuproptosis, while copper ions, especially Cu^+^, catalyze Fenton-like reactions with H_2_O_2_ in the TME, producing additional ˙OH and thereby potentiating overall oxidative stress. Finally, copper ions or SDT-induced ROS deplete GSH, which suppresses the antioxidant defense system and further sensitizes cells to oxidative damage.

In such multimodal nanomedicines, the dual role of copper ions—serving both as effective inducers of cuproptosis and as efficient catalysts for CDT—represents a highly economical and powerful design principle. Copper is typically loaded into nanomedicines in the form of Cu^2+^.^79,80^ Within the TME, particularly under conditions of high GSH levels, Cu^2+^ can be reduced to Cu^+^.^81–83^ Importantly, Cu^+^ is more effective than Cu^2+^ in inducing cuproptosis by directly binding to lipoylated proteins and in Fenton-like catalysis.^36,84–86^ As a result, the ROS created by SDT deplete GSH, further tilting the equilibrium towards the more toxic Cu^+^ state and enhancing oxidative stress.^43^ Consequently, the interaction between the nanomedicine and the TME drives the evolution of copper speciation and thereby directs the resulting therapeutic outcome—a balanced combination of cuproptosis and CDT.

### 3.2 Application in breast cancer

**Fig. 6:**
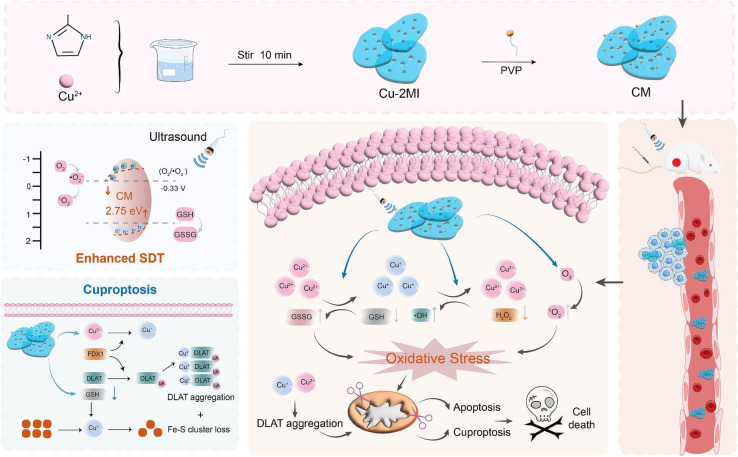
Schematic illustration of the CM. Reprinted with permission from ref. 87. Copyright 2025 Elsevier.

Zhong's group engineered a 2D copper-based piezoelectric metal organic frameworks (MOF), known as CM (Fig. 6), by coordinating copper with dimethylimidazole.^87^*In vitro*, GSH reduces Cu^2+^ ions in CM to Cu^+^, which then catalyzes the transformation of endogenous H_2_O_2_ into cytotoxic ˙OH through a Fenton-like reaction. This process simultaneously reduces GSH levels, which in turn increases oxidative stress. Cuproptosis is primarily induced by the released Cu^+^ ions, which directly bind to lipoylated DLAT and cause its abnormal oligomerization.^87^ Western blot and immunofluorescence analyses confirmed the oligomerization of DLAT and the downregulation of FDX1 and LIAS, key upstream regulators of the cuproptosis pathway. When combined with ultrasound, CM (CM+US) showed notable cytotoxic effects on 4T1 breast cancer cells. The treatment resulted in significant intracellular ROS and an apoptosis rate of almost 70%. In 4T1 tumor-bearing mice, the CM+US treatment achieved a tumor suppression rate as high as 90%, clearly outperforming CM alone (30% suppression) or US alone. The analysis of RNA sequencing data from tumor tissues in the CM+US group showed a decrease in anti-apoptotic gene expression and an increase in pro-apoptotic gene expression, along with changes in copper-related (such as *FDX1*, *DLAT*, *ATP7B*) and inflammation-associated genes (such as *Aim2*, *Nlrp3*). According to these findings, CM+US promotes cell death through different pathways, such as apoptosis, cuproptosis, and pyroptosis.

**Fig. 7:**
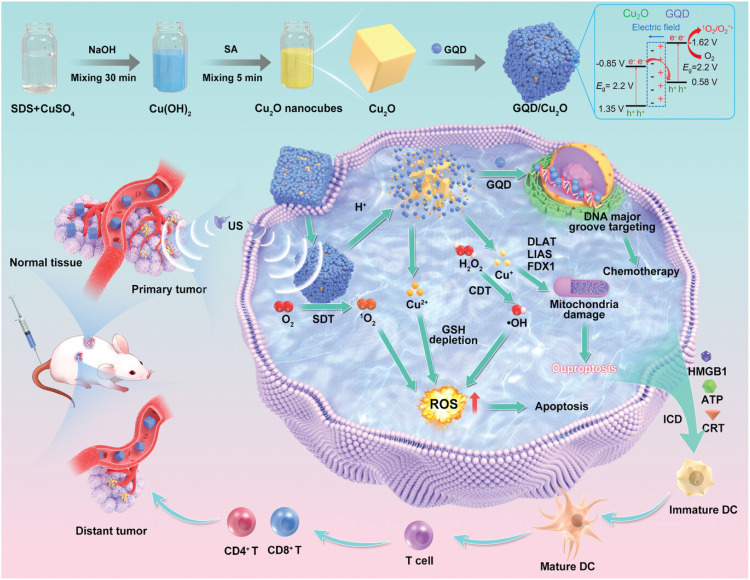
Schematic illustration of the GQD/Cu_2_O. Reprinted with permission from ref. 78. Copyright 2024 Wiley.

In another study, Yan *et al.* constructed a *Z*-scheme heterojunction (GQD/Cu_2_O) by depositing positively charged graphene quantum dots (GQDs) onto the surface of negatively charged Cu_2_O nanocubes *via* electrostatic adsorption (Fig. 7).^78^ The GQD layer acts as a protective barrier, preventing the non-tumor-specific degradation of Cu_2_O nanocubes and avoiding damage to normal tissues. Cu^+^ is released from the heterojunction degradation exclusively in the acidic TME. The GQD/Cu_2_O *Z*-scheme heterojunction significantly enhanced the US-triggered generation of ROS by promoting charge separation and suppressing electron–hole recombination. The ROS generation rate constant of GQD/Cu_2_O was higher than that of Cu_2_O alone. The GQD/Cu_2_O+US treatment effectively killed 4T1 cells and induced pronounced expression of cuproptosis markers (FDX1, DLAT).

While highly effective in 4T1 models, both platforms face challenges. The CM MOF's long-term biodegradability and potential off-target toxicity of copper ions are not fully addressed.^87^ Although the GQD/Cu_2_O heterojunction shows potential, its complex synthesis process could impede large-scale production and consistency between batches. Both studies did not thoroughly assess long-term systemic toxicity or degradation pathways, which is a significant barrier to clinical application.

### 3.3 Application in a glioblastoma model

**Fig. 8:**
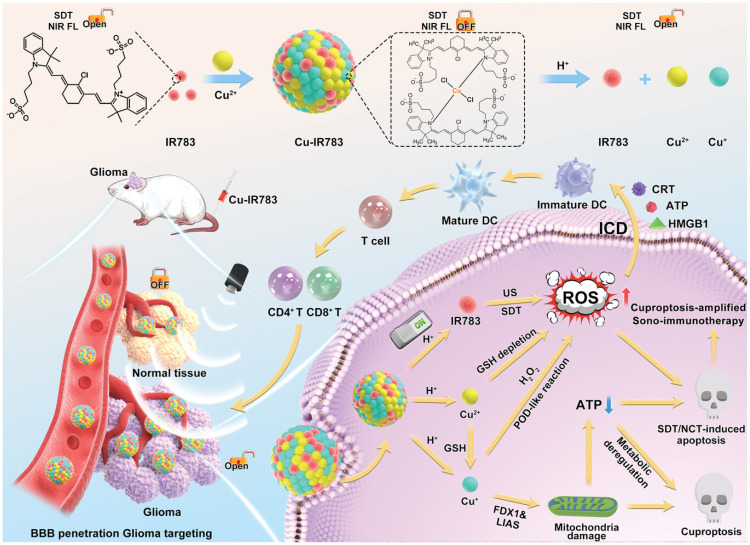
Schematic illustration of the Cu-IR783. Reprinted with permission from ref. 63. Copyright 2024 Wiley.

Treating GBM is challenging due to the presence of the blood–brain barrier (BBB) and the sensitivity of healthy brain tissue.^88–90^ A therapeutic agent that remains “off” during circulation and turns “on” only at the tumor site is highly desirable. Hu *et al.* constructed an intelligent nano-assembly (Cu-IR783 NPs) based on the coordination-driven self-assembly of the sonosensitizer IR783 with Cu^2+^ ions (Fig. 8). This nanoparticle exhibits a TME-responsive “off–on” near-infrared (NIR) imaging and therapeutic activity.^63^ In the acidic TME, the coordination bond between IR783 and Cu^2+^ breaks, leading to the dissociation of Cu-IR783 NPs and the release of IR783. This process restores the NIR fluorescence of IR783 and its sonosensitizing ability to generate ^1^O_2_ upon US irradiation. Cu-IR783 NPs demonstrated tumor-cell-specific activation and cytotoxicity in U87-MG glioblastoma cells, especially when combined with US and/or H_2_O_2_. In an orthotopic glioblastoma model, US assistance enhanced the BBB penetration of Cu-IR783 NPs. The Cu-IR783+US treatment group achieved complete tumor suppression, with a 100% survival rate at 50 days.

The acidic TME of GBM (pH < 7.0) provides the trigger for Cu-IR783 NP dissolution, which releases IR783 for NIR imaging and SDT, and copper ions for CDT/cuproptosis. US not only activates SDT but also can transiently open the BBB, improving nanoparticle delivery. The combination of these features makes this system highly effective in GBM therapy.

### 3.4 Application in melanoma

Melanoma is the most aggressive and lethal form of skin cancer. It originates from the malignant transformation of melanocytes and is characterized by high invasiveness, metastatic potential, and poor prognosis.^91–93^ Even though there have been major advances in therapies targeting important oncogenic mutations and immune checkpoints, low response rates and acquired resistance remain significant clinical issues.^93–95^ Therefore, it is important to develop a treatment strategy that provides multimodal synergy, precise targeting, and high safety.

**Fig. 9:**
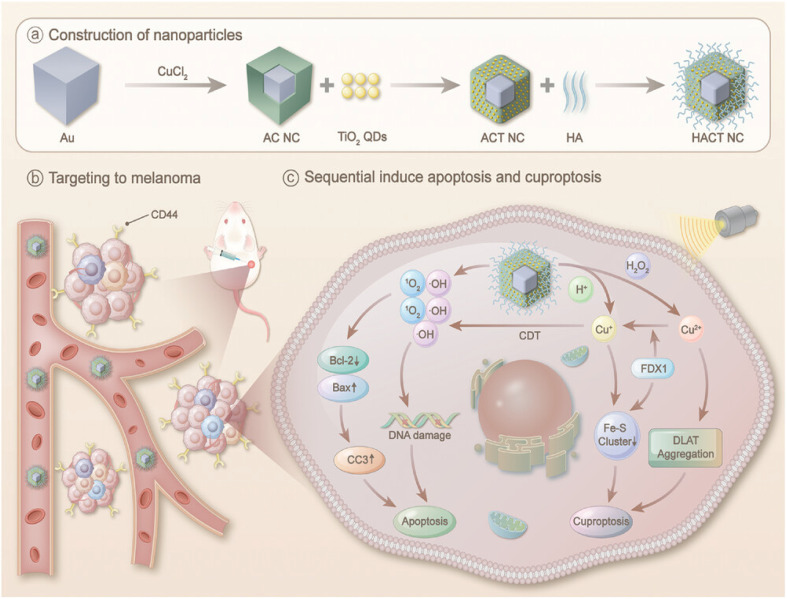
Schematic illustration of the HACT NCs. (a) Production of HACT NCs. (b) HACT NCs could target to melanoma. (c) Schematic illustration of the cuproptosis mechanisms of HACT NCs. Reprinted with permission from ref. 96. Copyright 2024 Wiley.

Gao's group engineered a ternary heterojunction nanocomposite (HACT NCs) to create a stimuli-responsive synergistic therapy for melanoma (Fig. 9).^96^ With ultrasound exposure, HACT NCs act as powerful sonosensitizers. The optimized band structure enhances electron–hole separation, resulting in the production of significant singlet oxygen (^1^O_2_) and hydroxyl radicals (˙OH), which causes strong oxidative stress. At the same time, within the TME, HACT NCs perform chemodynamic therapy by catalyzing Fenton-like reactions that convert H_2_O_2_ into highly toxic ˙OH. This increases oxidative stress further and is associated with the release of Cu^+^/Cu^2+^ ions, leading to excessive copper buildup and triggering cuproptosis. The mechanism involves copper ions being released and binding to lipoylated proteins (such as DLAT) in the tricarboxylic acid cycle. This causes abnormal oligomerization and lowers the expression of iron–sulfur cluster proteins (such as FDX1 and LIAS), ultimately activating the cuproptosis pathway.

There is a notable synergy between HACT NC-mediated sonodynamic therapy, chemodynamic therapy, and cuproptosis. *In vitro* studies revealed that the combination of HACT NCs, ultrasound, and H_2_O_2_ had a potent toxic effect on B16F10 melanoma cells, leading to increased intracellular ROS levels and the dissipation of mitochondrial membrane potential. At the same time, the treatment notably raised intracellular copper levels and reduced the expression of crucial cuproptosis proteins FDX1 and LIAS, confirming that cuproptosis occurred.

The combination of HACT NCs and ultrasound treatment resulted in nanoparticles accumulating specifically at the tumor site, markedly suppressing tumor growth and enhancing survival in the B16F10 tumor-bearing mice. Histological examination showed significantly higher ROS levels, reduced Ki-67 proliferation marker expression, increased cell death, and lower expression of cuproptosis-related proteins FDX1 and LIAS in the HACT NCs+US treatment group. Moreover, systemic toxicity tests revealed that HACT NCs are biocompatible and hemocompatible.

This research effectively developed a ternary heterojunction nanoplatform that simultaneously responds to US and the TME, synergistically inducing oxidative stress, chemodynamic therapy, and cuproptosis. This provides a groundbreaking strategy and experimental foundation for targeted melanoma therapy based on the induction of cuproptosis.

## 4. Sonodynamic nanomedicines for combined cuproptosis induction with immunotherapy

### 4.1 Principles of immunotherapy and its synergy with SDT-induced cuproptosis

The cooperation of SDT and cuproptosis not only directly and efficiently kills tumor cells but also more importantly, can induce a specific form of cell death known as ICD.^97–99^ As tumor cells die during ICD, they release a large number of damage-associated molecular patterns (DAMPs) like ATP, calreticulin (CRT), and HMGB1.^100–103^ These molecules direct dendritic cells (DCs) to infiltrate and become activated, thereby launching a potent and targeted immune response against tumors.^104–106^ This method of converting “cold” tumors into “hot” tumors effectively activates the host immune system to eliminate tumor cells at both primary and distant sites. Moreover, it reveals robust synergistic effects when combined with immune checkpoint blockade (ICB) therapy.

Both SDT and cuproptosis are potent ICD inducers, operating through complementary mechanisms: SDT generates acute oxidative stress, while cuproptosis imposes proteotoxic stress from mitochondrial dysfunction. The combination of these stresses creates a highly immunogenic form of cell death capable of converting immunologically “cold” tumors into “hot” tumors.

By combining SDT with cuproptosis across different nanomedicine platforms like MOFs, lactate dehydrogenases (LDHs), and heterojunctions, researchers have effectively induced ICD and stimulated antitumor immunity. SDT generates high ROS levels, and when combined with the specific stress signals from cuproptosis, they become powerful inducers of cellular stress, causing DAMPs to be released. As a result, this cascade promotes ICD and enhances immune responses against tumors.

### 4.2 Application in breast cancer

**Fig. 10:**
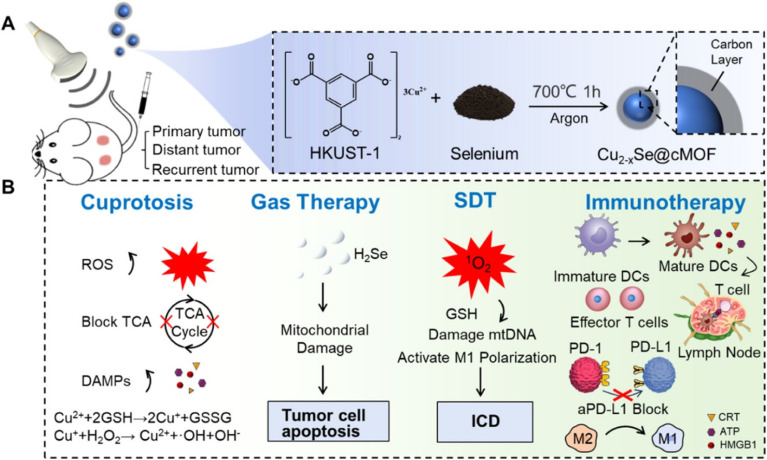
Schematic illustration of the Cu_2−*x*_Se@cMOF. (A) Production of Cu_2−_*_x_*Se@cMOF. (B) The dual enhancement of immune function by Cu_2−_*_x_*Se@cMOF. Reprinted with permission from ref. 67. Copyright 2024 American Chemical Society.

Zhenqi Jiang's team created a carbonized metal–organic framework (Cu_2−*x*_Se@cMOF) with diverse capabilities and linked it with an immune checkpoint inhibitor (anti-PD-L1 antibody).^67^ This nanoplatform releases copper ions under US irradiation, which specifically induce cuproptosis to kill tumor cells. The released copper ions, particularly Cu^+^, are believed to directly bind to the lipoylated proteins of the TCA cycle, leading to the aggregation of DLAT and the downregulation of FDX1 and LIAS, thereby initiating cuproptosis. Cuproptosis not only directly induces tumor cell apoptosis but also starts ICD, which leads to the release of DAMPs such as HMGB1 and CRT (Fig. 10). These signals then activate DCs and promote T cell infiltration. Moreover, the copper ions released from Cu_2−*x*_Se@cMOF can increase PD-L1 expression in tumor cells, thus boosting the effectiveness of anti-PD-L1 antibody-mediated immune checkpoint blockade. In both *in vitro* and *in vivo* environments, this combination therapy effectively induced tumor ICD. The combined strategy in 4T1 tumor-bearing mice effectively removed primary tumors and, by triggering a systemic antitumor immune response, significantly reduced the growth and metastasis of tumors located elsewhere. The findings indicate a robust therapeutic synergy when cuproptosis is combined with immunotherapy. This investigation presents a novel platform and mechanistic insights aimed at precision-enhanced cancer immunotherapy *via* cuproptosis.

### 4.3 Application in colorectal cancer

**Fig. 11:**
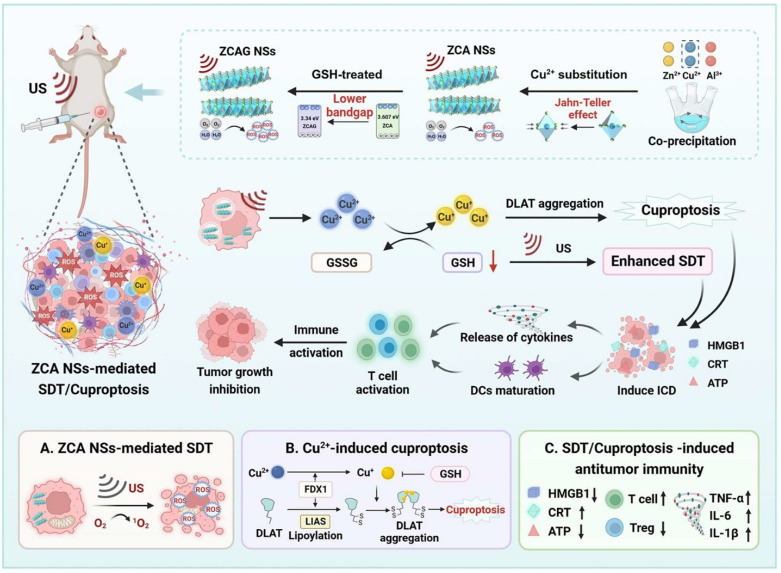
Schematic illustration of the ZCA NSs. (A) The sonodynamic activity of ZCA NSs can be enhanced by Cu^2+^. (B) ZCA NSs-induced copper overload effectively triggered cuproptosis. (C) ZCA NS-mediated SDT/cuproptosis anticancer therapy could trigger ICD. Reprinted with permission from ref. 107. Copyright 2024 American Chemical Society.

Tang *et al.* developed bioactive layered double hydroxide nanosheets (ZCA NSs) by substituting zinc in zinc–aluminum layered double hydroxides with copper (Fig. 11).^107^ Under US irradiation, ZCA NSs serve as efficient sonosensitizers. They generate high levels of ROS through Jahn–Teller-enhanced sonodynamic activity while simultaneously depleting GSH, thereby amplifying oxidative stress within tumor cells. Moreover, Cu^2+^ in ZCA NSs is reduced to Cu^+^ by intracellular GSH, and the resulting Cu^+^ further induces cuproptosis. Specifically, the accumulation of Cu^+^ binds directly to lipoylated proteins, such as DLAT, in the TCA cycle, causing abnormal oligomerization and a decrease in Fe S cluster proteins (such as FDX1 and LIAS), ultimately leading to proteotoxic stress and cell death.

ZCA NS-mediated sonodynamic therapy and cuproptosis notably work together effectively. *In vitro* studies indicated that the combination of ZCA NSs and ultrasound (ZCA NSs+US) showed potent cytotoxicity, resulting in increased ROS and ICD, characterized by CRT exposure, HMGB1 release, and enhanced ATP secretion. The maturation of DCs was significantly promoted by these DAMPs, which subsequently activated T cell immune responses.

The ZCA NSs+US treatment in both 4T1 and CT26 tumor-bearing mouse models not only fully eliminated the primary tumors but also effectively reduced metastasis to the lungs and liver. Western blot analysis showed a decrease in cuproptosis-related proteins like FDX1 and LIAS, while CLSM images indicated an increase in immune-related molecules such as HMGB1 and CRT in tumor cells. These findings reveal that the therapy, through the combined action of sonodynamic-induced cuproptosis, not only directly targets tumor cells but also activates antitumor immunity and transforms the immunosuppressive microenvironment. The study delivers significant experimental evidence supporting a combined treatment strategy of cuproptosis and immunotherapy.

### 4.4 Application in GBM

Recent GBM studies have primarily focused on direct treatment results. The immune mechanisms found in other tumors suggest that brain tumors might experience similar immunogenic activation. This crucially depends on whether the BBB permits immune cells to traffic effectively or DAMPs to be released from the tumor. Considering the immunologically ‘cold’ characteristics of GBM, this is an important area for additional research. Typically, GBM is known as an immunologically ‘cold’ tumor, obstructing efficient immune surveillance. Within the brain tumor, US-activated Cu IR783 nanoparticles trigger ICD and cuproptosis, serving as a crucial starting point for antitumor immunity activation.^63^ Research shows that the Cu^+^ ions released not only boost ROS production through Fenton-like reactions but also directly cause DLAT aggregation and mitochondrial dysfunction, resulting in cuproptosis. This method works together with sonodynamic/chemodynamic therapy, greatly enhancing the strength and immune response of ICD. Significantly, the cuproptosis and ROS burst triggered by Cu-IR783 NPs can collaboratively remodel the immunosuppressive tumor microenvironment, fostering DC maturation and boosting cytotoxic T cell infiltration. This provides a novel strategy to overcome GBM's immune evasion. Although this combined mechanism shows promise, there are still challenges, especially in enhancing the efficiency of T cell infiltration into brain tumors. Combining this approach with strategies like T-cell recruitment or immune checkpoint modulation holds potential for achieving local tumor suppression and distant immunotherapeutic effects against metastatic tumors.

### 4.5 Application in pancreatic cancer

**Fig. 12:**
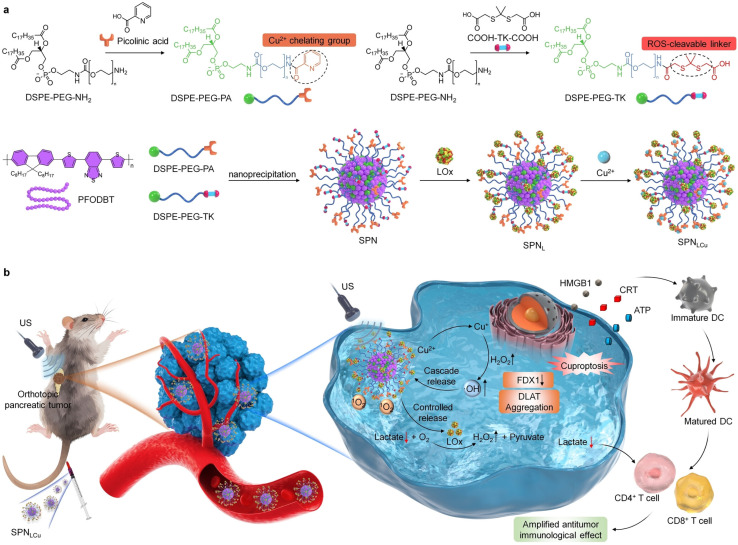
Schematic illustration of the SPNLCu. (a) The production of SPNLCu. (b) Schematic illustration of the antitumor mechanisms of SPNLCu. Reprinted with permission from ref. 108. Copyright 2024 Wiley.

Yu and colleagues created a semiconducting polymer nanoreactor (SPNLCu) by combining a semiconducting polymer with lactate oxidase (Fig. 12).^108^*In vitro* studies revealed that the combination of SPNLCu and ultrasound significantly harmed pancreatic cancer cells. SPNLCu+US treatment led to a significant increase in intracellular ROS and an ICD rate nearing 70%, with little toxicity to healthy cells.

The treatment with SPNLCu+US significantly inhibited tumor growth in mice with pancreatic tumors. The treatment showed greater efficacy against both subcutaneous and deep-tissue orthotopic pancreatic tumors compared to using SPNLCu or US alone.

Moreover, Cu^+^-induced cuproptosis resulted in ICD, which in turn activated antitumor immunity. This approach synergistically destroys tumor cells and boosts therapeutic effectiveness through the combined efforts of cuproptosis and immune activation.

All platforms induce ICD by generating ROS (from SDT) and causing proteotoxic stress (from cuproptosis). However, the Cu_2−*x*_Se@cMOF introduces a unique third mechanism: gas therapy (H_2_Se), which further contributes to cell stress and ICD.^67^ The ZCA NSs leverage the Jahn–Teller effect, a specific electronic distortion from Cu^2+^ substitution, to enhance sonodynamic ROS production, demonstrating a materials-science-driven approach to boost ICD.^107^ Meanwhile, all platforms show an abscopal effect in bilateral tumor models. The Cu_2−*x*_Se@cMOF + anti-PD-L1 combination is particularly potent, demonstrating not only primary tumor eradication and distant tumor suppression but also long-term immune memory, preventing tumor rechallenge.^67^

## 5. Sonodynamic nanomedicines for combined cuproptosis induction with other physical and metabolic therapies

### 5.1 Combined gas therapy

Gas therapy is an emerging anticancer strategy that relies on bioactive “gasotransmitters”—such as NO, H_2_S, CO, or H_2_Se—that exist endogenously or can be delivered exogenously.^109–111^ At low concentrations, these molecules regulate normal physiological functions. However, at high levels or within the specific TME, they can be converted into potent cytotoxic agents that induce cancer cell death. As described, the multifaceted carbonized metal–organic framework nanoplatform (Cu_2−*x*_Se@cMOF) exhibits excellent performance for precise, US-controlled release of multiple therapeutics.^67^ Under US irradiation, this material simultaneously accomplishes three functions: (1) generating reactive oxygen species for SDT; (2) releasing copper ions to induce cuproptosis; (3) releasing the therapeutic gas H_2_Se for gas therapy. The triple synergy of cuproptosis, SDT, and gas therapy, together with immune checkpoint inhibition, allows Cu_2−*x*_Se@cMOF+US to induce tumor cell death through several pathways (including apoptosis and cuproptosis) and stimulate a systemic antitumor immune response.

**Fig. 13:**
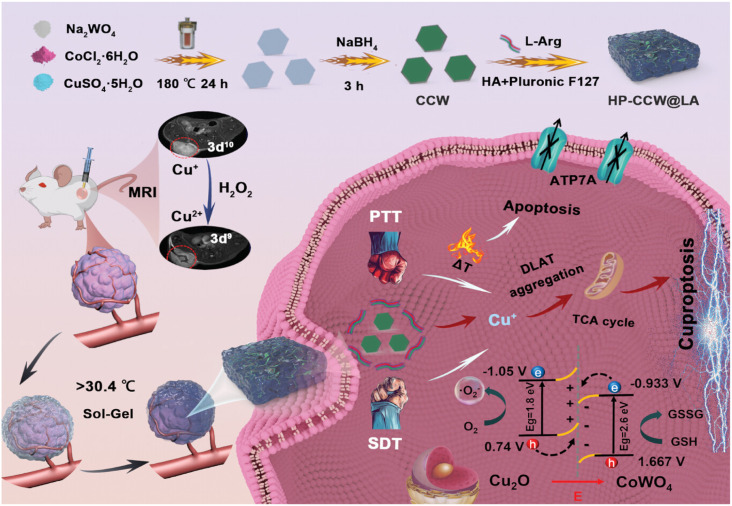
Schematic illustration of the HP-CCW@LA. Reprinted with permission from ref. 112. Copyright 2024 Wiley.

Moreover, Yang *et al.* developed a tumor-cuproptosis platform that combines gas therapy with photothermal therapy (PTT) by creatively synthesizing a closely-contacted Cu_2_O–CoWO_4_ nanosheet heterojunction (HP-CCW@LA) (Fig. 13).^112^l-Arginine (L-Arg) acted as a crucial reducing agent, driving the controlled release of highly active Cu^+^ and breaking down to generate NO gas after treatment. This gas molecule shows considerable bioactivity, significantly boosting therapeutic effects. Importantly, L-Arg's decomposition was enhanced by HP-CCW@LA in the TME: HP-CCW@LA combined with endogenous hydrogen peroxide to transform surface Cu^+^ into Cu^2+^. This valence change provided MRI capabilities to the system and also modified the TME by generating NO gas, allowing for real-time assessment of the therapeutic effect.

### 5.2 Combined PTT

PTT represents a cancer treatment method that is minimally invasive and relies on materials with excellent photothermal conversion capabilities.^113–115^ These nanoparticles accumulate at the tumor site and, when precisely irradiated with an external light source, effectively transform light energy into heat.^116–118^ This leads to a quick increase in local temperature (generally over 42 °C), resulting in protein denaturation, membrane disruption, and irreversible thermal damage within cancer cells.^119,120^ Finally, it can achieve precise tumor elimination with strong control and minimal damage to nearby normal tissues.

As previously described, CCW-NH is a fundamental breakthrough by organically combining the gas-therapeutic role of L-Arg with the benefits of PTT, significantly boosting antitumor effectiveness.^112^ HP-CCW@LA showed remarkable photothermal conversion capabilities, with a temperature elevation of 41.0 °C when exposed to 655 nm laser light at 1 mg mL^−1^, and a high efficiency of 24.51%. The photothermal property, combined with a *Z*-scheme charge transfer mechanism, led to a 1.7-fold increase in copper ion release when subjected to both US and NIR laser irradiation. This significantly boosted cuproptosis compared to untreated groups. In 4T1 tumor-bearing mice, combining HP-CCW@LA with ultrasound and NIR laser irradiation led to a tumor suppression rate reaching 95.1%, which was much more effective than using HP-CCW@LA alone (∼40% suppression) or ultrasound/laser alone. It was evidenced that the synergy resulted from the interaction of PTT and SDT in enhancing copper ion release, with NO gas from L-Arg intensifying oxidative stress and TME modulation.

### 5.3 Combined ferroptosis therapy

Ferroptosis therapy is a method for treating tumors by triggering iron-dependent lipid peroxidation to kill cancer cells.^121–123^ The primary mechanism takes advantage of cancer cells' high need for iron. Raising the concentration of ferrous ions (Fe^2+^) inside the cells initiates Fenton reactions that generate a large amount of ROS. It also suppresses key antioxidant defense systems such as glutathione peroxidase 4 (GPX4).^124–126^ This results in a toxic buildup of lipid peroxides on cell membranes, eventually damaging the membrane structure and leading to cell death.^127,128^ This approach is particularly suitable for treating tumors that are resistant to conventional radiotherapy or chemotherapy or avoid apoptosis, due to its unique characteristics.

**Fig. 14:**
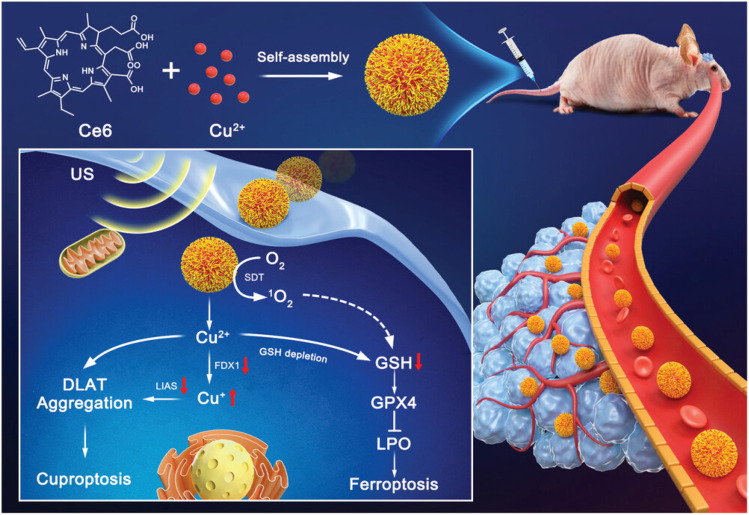
Schematic illustration of the Ce6@Cu NPs. Reprinted with permission from ref. 129. Copyright 2024 Wiley.

Yang Zhu's research group achieved the development of a self-assembled, carrier-free nanosensitizer (Ce6@Cu NPs) through the coordination between Cu^2+^ ions and chlorin e6 (Ce6) as the sonosensitizer (Fig. 14).^129^ The design smartly allows for the simultaneous induction of ferroptosis and cuproptosis, providing a new method for GBM treatment. Cu^2+^ released from Ce6@Cu NPs in the TME is transformed into the more toxic Cu^+^ by GSH. The lipoylated protein DLAT in mitochondria is directly bound by Cu^+^, which induces abnormal oligomerization and significantly decreases the expression of key cuproptosis regulators FDX1 and LIAS, resulting in proteotoxic stress and the induction of cuproptosis. Moreover, Ce6@Cu nanoparticles effectively deplete intracellular GSH, leading to the inhibition of the key antioxidant enzyme GPX4, promoting the accumulation of lipid peroxides and triggering ferroptosis. Ultrasound irradiation enhances the effect by triggering Ce6 to produce ROS, which in turn promotes GSH consumption and lipid peroxidation. US not only enhances the sonodynamic effect of Ce6 but also causes lysosomal rupture, which speeds up the escape of nanoparticles into the cytoplasm, thereby increasing the efficiency of Cu^+^ release and its reaction with GSH. This results in a triple synergistic effect known as “sonodynamic ferroptosis cuproptosis”. Ce6@Cu nanoparticles showed outstanding ability to penetrate the BBB, leading to substantial accumulation in orthotopic glioblastoma and overcoming the delivery challenge in tumor treatment. Ultrasound serves as an external stimulus that enables precise spatiotemporal control over treatment, activating therapeutic effects only in the specific area that is irradiated. Through a carrier-free self-assembly strategy, this study successfully constructed a multifunctional nanoplatform integrating sonodynamic activation, GSH depletion, and copper ion delivery. It achieved for the first time the efficient synergy of ferroptosis and cuproptosis driven by sonodynamics. This mechanism of ‘dual death’ synergy provides a safe, precise, and novel therapeutic strategy for GBM, creating new research opportunities in oncology.

### 5.4 Combined metabolic therapy

Metabolic therapy is a method designed to prevent tumor development by focusing on the unique metabolic changes in cancer cells.^130–132^ Its primary principle is based on the basic differences in how energy is acquired by tumor cells compared to normal cells.^133,134^ By employing specific drugs to disrupt the uptake and use of glucose, glutamine, and lipids by cancer cells, we can effectively cut off the “fuel” supply of tumors.^135^ This results in the death of cancer cells through energy depletion and inhibited biosynthesis. Moreover, modulating the metabolic condition of the TME can relieve immunosuppression. This turns metabolic therapy into a crucial adjuvant that greatly improves the effectiveness of radio-/chemotherapy and immunotherapy.

Yu's team introduced an innovative approach (SPNLCu) for treating pancreatic cancer by accurately combining cuproptosis with metabolic therapy.^108^ GSH reduces chelated Cu^2+^ to Cu^+^ in the TME. The release of LOx from SPNLCu, which is conjugated with lactate oxidase through a ROS-cleavable linker, occurs under ultrasound irradiation (Fig. 12). In the TME, LOx converts high concentrations of lactate into pyruvate and simultaneously generates H_2_O_2_. The reduction of lactate levels in the TME through lactate depletion helps to alleviate lactate-mediated immunosuppression, thereby creating a more favorable environment for immunotherapy. Simultaneously, the H_2_O_2_ that is produced reacts with Cu^+^, increasing the cuproptosis effect. In mouse models of pancreatic cancer, SPNLCu+US treatment resulted in significant suppression of tumors. Analysis of tumor tissues revealed that the SPNLCu+US group had the lowest lactate levels, the most significant downregulation of FDX1, the most pronounced DLAT oligomerization, and increased immune cell infiltration. This confirmed the synergistic effect of metabolic modulation and cuproptosis. This investigation is the first to achieve US-triggered, enhanced lactate depletion coupled with precise cuproptosis induction, providing a novel strategy for pancreatic cancer therapy.

Metabolic interventions such as lactate depletion are highly innovative as they directly address TME-induced drug resistance and immunosuppression. Future studies should target the development of multi-responsive systems that can intelligently sequence or apply these modalities concurrently, using TME cues for the best synergy.

Overall, combining SDT–cuproptosis with other approaches such as ferroptosis, gas therapy, and metabolic therapy involves specific material requirements and constraints. The major advantage of Ce6@Cu NPs its “carrier-free” nature, which simplifies synthesis and regulatory approval. However, the lack of a targeting moiety may lead to off-target copper toxicity. HP-CCW@LA provides excellent local retention and sustained release. However, the system is complex, incorporating multiple synthetic components, which complicates manufacturing, quality control, and safety assessment. SPNLCu uniquely addresses the TME by depleting lactate, which not only removes an immunosuppressive metabolite but also fuels the therapeutic reaction. Nonetheless, its limitation is reliance on endogenous H_2_O_2_ levels, which can vary significantly between tumors, potentially leading to unpredictable efficacy.

## 6. Conclusion and future perspectives

The synergy between SDT and cuproptosis introduces a new model in cancer nanomedicines, offering a multifaceted treatment strategy that overcomes the limitations of each individual approach. In conclusion, the integration of SDT and cuproptosis has been accomplished through the strategic design of nanomedicines. These include metal–organic frameworks (MOFs), heterojunctions, layered double hydroxides, porous organic polymers, and biomimetic nanoparticles. These innovative nanomedicines display outstanding antitumor efficacy in various tumor models, including breast cancer, glioblastoma, colorectal cancer, melanoma, pancreatic cancer, lymphoma, and osteosarcoma (Table 1).

**Table 1:** Summary of representative nanoplatforms for SDT and cuproptosis combination therapy

Nanoplatform	Nanomaterial type	Tumor model	Therapeutics	Ref.
CRUPPA19	MOF (UiO-66–NH_2_)	B-cell lymphoma	Cuproptosis, apoptosis, autophagy, ICD	66
Cu/ART@Hpy	Porous organic polymer (POP)	4T1 breast cancer	Cuproptosis, apoptosis, CDT	68
CM	2D MOF	4T1 breast cancer	Cuproptosis, pyroptosis, apoptosis	87
GQD/Cu_2_O	*Z*-Scheme heterojunction	4T1 breast cancer	Cuproptosis, apoptosis, chemotherapy	78
Cu-IR783 NPs	Coordination-driven nano-assembly	U87-MG glioblastoma	Cuproptosis, CDT	63
HACT NCs	Ternary heterojunction	B16F10 melanoma	Cuproptosis, CDT	96
ZCA NSs	Layered double hydroxide (LDH)	4T1, CT26 tumors	Cuproptosis, immunotherapy	107
Cu_2−*x*_Se@cMOF	Carbonized MOF	4T1 breast cancer	Cuproptosis, apoptosis, gas therapy	67
SPNLCu	Semiconducting polymer nanoreactor	Pancreatic cancer	Cuproptosis, metabolic therapy, immunotherapy	108
HP-CCW@LA	Nanosheet heterojunction	4T1 breast cancer	Cuproptosis, apoptosis, PTT, gas therapy	112
Ce6@Cu NPs	Carrier-free self-assembly	U87-MG glioblastoma	Cuproptosis, ferroptosis	129

The cooperative mechanism between SDT and cuproptosis involves various interdependent pathways that converge on mitochondria and the cellular redox state. Firstly, ultrasound-induced sonosensitizers generate ROS, which directly cause tumor cell apoptosis. This ROS burst also lowers intracellular GSH levels, a key copper chelator, thereby increasing the toxic copper ions and sensitizing cancer cells to cuproptosis. This sensitization is crucial as cuproptosis is initiated by the binding of Cu^+^ to lipoylated TCA cycle proteins, a process dependent on FDX1 to generate Cu^+^ from Cu^2+^. The subsequent oligomerization of DLAT, a direct consequence of this binding, leads to proteotoxic stress and is a hallmark of cuproptosis.^32,33,37^ Therefore, the ROS from SDT do not only function as a sensitizer by lowering GSH. They actively disrupt mitochondrial metabolic integrity and downregulate the mechanisms (FDX1/LIAS) that manage the cell's sensitivity to copper-induced mortality. This breakdown in metabolism allows copper ions to more easily attach to the remaining lipoylated proteins like DLAT, resulting in cuproptosis. For instance, Yan *et al.* demonstrated that GQD/Cu_2_O achieve cascade amplification of ROS production through Cu^+^-mediated Fenton-like reactions and Cu^2+^-facilitated GSH depletion, simultaneously triggering cuproptosis *via* DLAT oligomerization and mitochondrial dysfunction.^78^ Secondly, copper ions released from nanomedicines, such as Cu_2_O nanocubes, Cu-doped MOFs, or Cu-containing layered double hydroxides, attach directly to lipoylated elements of the TCA cycle, causing proteotoxic stress and ICD. Significantly, Tang and colleagues found that copper-substituted ZnAl layered ZCA NSs take advantage of the Jahn–Teller effect to enhance sonodynamic activity, deplete GSH, and trigger cuproptosis, resulting in strong antitumor immunity.^107^ Third, the synergy between SDT and cuproptosis induces an ICD effect that enhances DC maturation, increases cytotoxic T lymphocyte infiltration, and reverses the immunosuppressive tumor microenvironment, as shown by several studies highlighting a strong abscopal effect in bilateral tumor models.

Recent breakthroughs in nanomedicine engineering have markedly boosted the effectiveness of SDT–cuproptosis combination therapy. Ce6@Cu nanoparticles have successfully induced both ferroptosis and cuproptosis by enhancing lipid peroxidation through sonodynamics and causing copper overload, showing remarkable ability to penetrate the BBB for treating glioblastoma.^129^ Biomimetic strategies, exemplified by Chen *et al.*'s SonoCu and Li *et al.*'s dual-responsive CytoNano, leverage neutrophil membrane coating and acid-responsive polymers to achieve tumor-specific accumulation and mitochondria-targeted delivery, minimizing off-target toxicity while maximizing therapeutic efficacy.^65^

Even with these encouraging developments, there are still significant challenges that need to be overcome for clinical translation. Firstly, synthesizing complex multifunctional nanomedicines on a large scale while following GMP guidelines remains a technical challenge. The structural complexity of these nanoplatforms—often consisting of multiple components such as sonosensitizers, copper sources, targeting ligands, and stimuli-responsive coatings—creates significant challenges for quality control, batch-to-batch consistency, and regulatory approval. In addition to this challenge, the clinical use of these agents is severely limited by two primary factors: unresolved questions regarding their long-term biosafety and the fundamental constraints of US physical fields. The long-term presence of copper-based nanomaterials *in vivo* is a considerable biosafety concern. In mouse models, acute toxicity studies usually show positive biocompatibility. Nonetheless, several studies shown that these nanomedicines preferentially gather in the liver and spleen because of the EPR effect.^50,51,136^ For instance, Zhao *et al.* observed that Cu_2−*x*_Se@cMOF nanoparticles primarily accumulated in the liver and spleen, and while cleared over seven days, the long-term effects of residual copper and other metals on organ function remain unknown.^67^ The potential chronic toxicity, including hepatotoxicity, nephrotoxicity, and neurotoxicity from long-term copper overload, has not been systematically evaluated in large animal models. Second, the clinical application of US for SDT faces practical physical and technical limitations that are often underappreciated in preclinical studies. Even though US penetrates tissues more effectively than light, its performance is heavily dependent on the acoustic nature of the intervening tissues. The presence of bone and organs (such as lungs, gastrointestinal tract) severely attenuates or reflects US waves, creating acoustic shadowing that limits the treatment of deeply tumors.^108^ Moreover, the preclinical ultrasound parameters currently vary significantly across different studies.^63,78,87^ These parameters include frequency, intensity, duty cycle, and duration.^107^ Standardized protocols for activating different sonosensitizers remain lacking. Thus, accurately controlling the timing and location of US energy deposition to ensure predictable cuproptosis and ROS production in deep tumors is a significant engineering challenge. Addressing these issues requires moving away from preclinical studies focused on efficacy towards robust safety and translational research. Future designs of nanomedicine need to emphasize the importance of being biodegradable and safely cleared. Concurrently, the field must establish standardized US treatment protocols. Third, tumor heterogeneity presents a significant challenge, as the expression levels of cuproptosis-related proteins (such as FDX1, LIAS, and DLAT) and the metabolic dependency on mitochondrial respiration vary significantly across tumor types and individual patients. Selecting patients based on biomarkers will be crucial to determine who will benefit most from the SDT–cuproptosis combination therapy.

Looking forward, it is important to investigate multiple transformative directions. Artificial intelligence (AI) and machine learning approaches can accelerate the rational design of next-generation sonosensitizers by predicting optimal band structures, piezoelectric coefficients, and copper coordination geometries. High-throughput screening coupled with computational modeling may identify novel copper-based nanomedicines with enhanced sonodynamic activity and tumor-specific degradation profiles. Second, the integration of multi-omics technologies—including transcriptomics, proteomics, and metabolomics—will reveal the complex regulatory networks regulating cuproptosis sensitivity and resistance. The use of single-cell sequencing on tumors treated with SDT–cuproptosis nanomedicines can reveal dynamic changes in the immune microenvironment of the tumor and identify mechanisms of adaptive resistance. Third, advanced delivery systems such as exosome-mimetic nanovesicles, cell membrane-coated nanoparticles, and hydrogel-based implants offer unprecedented opportunities for spatiotemporally controlled drug release and reduced systemic toxicity. For instance, previous studies have reported an implantable oxidized bacterial cellulose membrane (TB/αPD-1@AuNCs/OBC) proving the potential of localized, sustained delivery for postoperative immunotherapy.^137^ Fourth, the deep integration of SDT–cuproptosis with emerging therapeutic modalities—including ferroptosis, pyroptosis, metabolic interventions, and oncolytic virotherapy—may yield synergistic effects beyond the additive effects of individual treatments. The simultaneous activation of multiple programmed cell death pathways could overcome tumor adaptive resistance mechanisms and elicit robust, long-lasting antitumor immunity.

In conclusion, the nanomedicine-enabled synergy between SDT and cuproptosis, enhanced by synergistic modalities such as chemodynamic therapy, immunotherapy, photothermal therapy, gas therapy, and metabolic interventions, establishes a powerful and precise antitumor paradigm. The wide variety of nanomedicines created offers a valuable foundation for exploring mechanisms and enhancing therapies. While significant challenges remain in biocompatibility assessment, scalable manufacturing, and clinical translation, the foundational research summarized in this review provides a robust basis for translating these innovative strategies from bench to bedside. With further interdisciplinary collaboration among material scientists, oncologists, immunologists, and translational researchers, SDT–cuproptosis nanomedicines are prepared to become a clinically effective option, offering new hope to patients with therapy-resistant and metastatic cancers.

## Conflicts of interest

The authors declare no conflict of interest.

## Data Availability

No primary research results, software or code have been included and no new data were generated or analysed as part of this review.
